# Partial Inhibition of mTORC1 in Aged Rats Counteracts the Decline in Muscle Mass and Reverses Molecular Signaling Associated with Sarcopenia

**DOI:** 10.1128/MCB.00141-19

**Published:** 2019-09-11

**Authors:** Giselle A. Joseph, Sharon X. Wang, Cody E. Jacobs, Weihua Zhou, Garrett C. Kimble, Herman W. Tse, John K. Eash, Tea Shavlakadze, David J. Glass

**Affiliations:** a Novartis Institutes for Biomedical Research, Cambridge, Massachusetts, USA

**Keywords:** aging, mTOR, mTORC1, muscle, rapalog, rapamycin, sarcopenia, skeletal muscle

## Abstract

There is a lack of pharmacological interventions available for sarcopenia, a progressive age-associated loss of muscle mass, leading to a decline in mobility and quality of life. We found mTORC1 (mammalian target of rapamycin complex 1), a well-established positive modulator of muscle mass, to be surprisingly hyperactivated in sarcopenic muscle. Furthermore, partial inhibition of the mTORC1 pathway counteracted sarcopenia, as determined by observing an increase in muscle mass and fiber type cross-sectional area in select muscle groups, again surprising because mTORC1 signaling has been shown to be required for skeletal muscle mass gains in some models of hypertrophy. Additionally, several genes related to senescence were downregulated and gene expression indicators of neuromuscular junction denervation were diminished using a low dose of a “rapalog” (a pharmacological agent related to rapamycin). Therefore, partial mTORC1 inhibition may delay the progression of sarcopenia by directly and indirectly modulating multiple age-associated pathways, implicating mTORC1 as a therapeutic target to treat sarcopenia.

## Abstract (precis)

There is a lack of pharmacological interventions available for sarcopenia, a progressive age-associated loss of muscle mass, leading to a decline in mobility and quality of life. We found mTORC1 (mammalian target of rapamycin complex 1), a well-established positive modulator of muscle mass, to be surprisingly hyperactivated in sarcopenic muscle.

## INTRODUCTION

Skeletal muscle size is physiologically regulated by load and activity and can decrease when loads are reduced. Muscle also atrophies, or decreases in size, under pathological conditions such as cancer, immobilization, and denervation (1). One setting where muscle mass and function are diminished is old age. This loss of muscle is called sarcopenia, and it is associated with a decrease in the ability to move, leading to morbidity and ultimately to mortality (2); indeed, a decrease in walking speed is one of the strongest predictors of mortality in humans, and this finding is associated with sarcopenia (3, 4). In addition to frailty and sarcopenia, aging of course affects every tissue system and greatly increases susceptibility to other serious diseases and comorbidities, such as cancer, heart failure, chronic kidney disease, loss of vision, dementia, and Alzheimer’s disease (1, 5, 6).

Experimental data strongly suggest the coordinated regulation of aging by distinct molecular pathways (7); modulation of these pathways can counteract several age-related diseases and comorbidities and can prolong life (7–10). Among these signaling pathways, genetic or pharmacological inhibition of mammalian target of rapamycin complex 1 (mTORC1) is thus far the best-validated intervention to delay age-related pathophysiological changes (11). For instance, the use of an mTORC1 inhibitor, rapamycin, even when administered at later stages in life, has been shown to extend life span in mice (12–15). Pharmacological agents related to rapamycin are called “rapalogs.” Use of a rapalog for age-related indications has recently been translated to human beings, where it was shown to improve responses to vaccinations in the elderly, coincident with decreasing signs of immune senescence (16). The low-dose rapalog treatment used in the human study was reverse-translated to rats, where it was shown that intervention late in life could prevent signs of age-related kidney pathology (17). However, there has always been concern about the potential effects of rapamycin and rapalogs on skeletal muscle. For example, inhibition of the mTORC1 pathway was shown to entirely block responses to compensatory hypertrophy in mice (18). This certainly gave the impression that activation of mTORC1 signaling was desirable for the maintenance of muscle mass. Most recently, it was shown that rapamcycin treatment inhibited muscle mass increase caused by myostatin loss (19). Thus, it seemed reasonable that inhibition of the pathway was not desirable in settings of muscle loss (1, 18, 20).

As to the pathway, Akt induces protein synthesis in part by activation of mTORC1 signaling (18, 21). mTOR exists in the distinct complexes mTORC1 and mTORC2. mTORC1 is characterized by the presence of RAPTOR (regulatory-associated protein of mTOR) (22), while TORC2 binds to RICTOR (rapamycin-insensitive partner of mTOR) (23, 24). The mTORC1 complex induces downstream signaling responsible for protein synthesis through phosphorylation and activation of S6 kinase 1 (S6K1) and via inhibition of 4EBP1 (24, 25) and is sensitive to inhibition by rapamycin and rapalogs. In addition to the anabolic function, Akt also limits muscle protein degradation and atrophy by phosphorylating and thereby inhibiting the FOXO (also known as Forkhead) family of transcription factors. Activation of FOXO3 is sufficient to induce atrophy (26, 27); transgenic expression of FOXO1 also leads to an atrophic phenotype (28, 29). FOXO1 and FOXO3 proteins transcriptionally upregulate expression of the muscle atrophy-associated E3 ligases, muscle RING finger 1 (MuRF1), and muscle atrophy F-box (MAFbx)/atrogin-1 (30–32). Both MuRF1 and MAFbx/atrogin-1 are specifically upregulated under atrophic conditions (33, 34) and target proteins that are critical for muscle structure and protein synthesis for degradation, thereby inducing muscle loss (35–37). mTORC1 itself can directly inhibit catabolic functions such as autophagy (38). Autophagy has been shown to promote muscle atrophy in response to fasting conditions in young animals (39). However, it was found that autophagy function is impaired in the aged and plays a role in age-related dysfunction in several different tissues (40, 41). Indeed, recent experimental evidence suggests that the restoration of autophagic flux in aged animals may prevent loss of muscle mass and function related to sarcopenia (42, 43).

mTORC1 inhibition has been widely suggested as a way to improve function in the elderly in various tissues. However, its potential as a therapeutic intervention for the treatment of sarcopenia has not been considered. Upon experimentation, we were surprised to learn that mTORC1 signaling is upregulated rather than downregulated coincident with signs of sarcopenia in rats; therefore, we explored the effects of rapalog treatment in this setting. The results demonstrate that the partial inhibition of mTORC1 is helpful in preventing pathological changes related to sarcopenia in select muscles.

## RESULTS

### Increased activation of the mTORC1 pathway with age.

**FIG 1:**
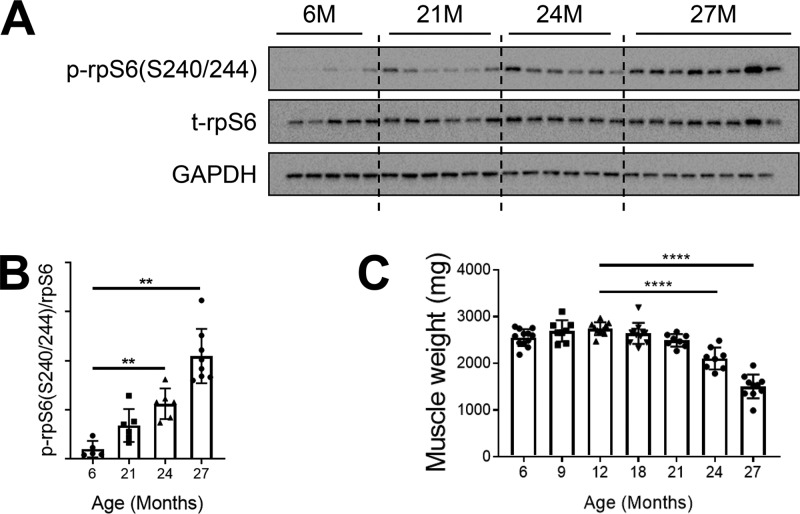
mTORC1 signaling is hyperactivated in sarcopenic skeletal muscle. (A) Immunoblots for phosphorylated (p) and total (t) protein for rpS6 in gastrocnemius muscles of rats aged 6, 21, 24, and 27 months (6M, 21M, 24M, and 27M, respectively) (*n* = 5 to 8). Glyceraldehyde-3-phosphate dehydrogenase (GAPDH) is shown as a protein loading control. (B) p-rpS6(S240/244) protein amounts were quantified relative to the respective total rpS6 protein amounts by densitometry. (C) Gastrocnemius muscle weights in rats aged 6, 9, 12, 18, 21, 24, and 27 months (*n* = 8 to 12). Data are means ± standard deviations of the means. Statistical significance was determined by a one-way ANOVA followed by Dunnett’s multiple-comparison tests. Means of results from all groups were compared to the mean of results from 12-month-old animals. Asterisks are used to denote significance as follows: **, *P* < 0.01; ****, *P* < 0.0001. The *y*-axis data in panel B represent arbitrary units.

Given prior reports that mTORC1 inhibition was helpful to treat a variety of age-related disorders but also data indicating that mTORC1 activation is required for muscle hypertrophy, we conducted a time course analysis of the mTORC1 pathway to examine the full scope of how its activity changes with age. In laboratory settings, Sprague-Dawley rats have an average life span of up to 2.5 to 3 years (44). In our study, male rats that were 6 months to 27 months of age were used. Protein lysates from gastrocnemius muscles were probed for the downstream effector of mTORC1, phosphorylated ribosomal protein S6 (rpS6), as a determinant of pathway activity. Basal (6-h-fasted) levels of phosphorylated rpS6 gradually increased as the rats aged, with a substantial increase of about 10-fold in the oldest animals (aged 27 months) compared with those aged 6 months (Fig. 1A and B). The age-related increase in mTORC1 signaling coincided with a decrease in muscle mass. Gastrocnemius muscle weights declined at 18 months and progressively atrophied at each later time point (Fig. 1C). Though muscle loss at that age is not a surprise, the coincidence of this loss with mTORC1 activation was quite unexpected, given that it favors muscle growth and hypertrophy.

### The RAD001 rapalog inhibits mTORC1 activity in sarcopenic muscle.

Experimental evidence shows that the use of rapalogs as therapeutic agents is beneficial in extending life span and counteracting age-related morbidities in humans and other evolutionarily diverse species (reviewed in reference 45). Despite evidence of similarly sustained mTORC1 signaling in aged muscle, its inhibition has not yet been studied in the context of sarcopenia.

We sought to determine whether rapalog treatment could counter the pathophysiological changes associated with sarcopenia. Aging rats display signs of sarcopenia beginning at 18 months (46). In the present study, aged rats (22 months) were dosed daily with vehicle or with RAD001 at 0.15 mg/kg of body weight or 0.5 mg/kg of body weight (here referred to as low-dose [LD] and high-dose [HD] RAD001, respectively) for 6 weeks. LD RAD001 is equivalent to a clinical dose of 0.5 mg in humans, ensuring therapeutic relevance (16). Vehicle-treated young adult rats (7 months) served as a comparative baseline for aging effects. At the end of the treatment, aged and young adult rats were 24 months and 9 months old, respectively, and are referred to here as such.

**FIG 2:**
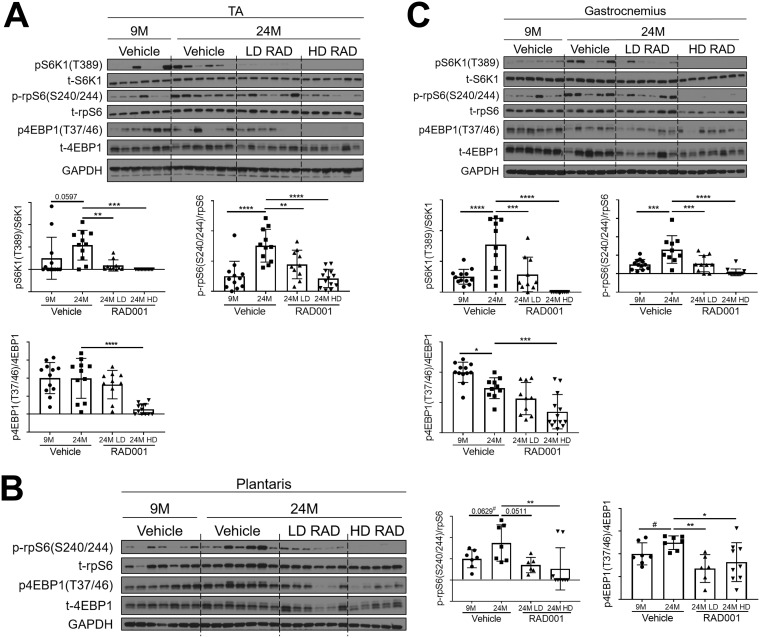
Confirmed mTORC1 inhibition following rapalog treatment. Representative immunoblots are shown for phosphorylated (p) and total (t) protein for S6K1, rpS6, and 4EBP1 in tibialis anterior (A), plantaris (B), and gastrocnemius (C) muscles of 9- and 24-month-old rats treated with vehicle and of 24-month-old rats treated with 0.15 mg/kg (LD) or 0.5 mg/kg (HD) RAD001. Glyceraldehyde-3-phosphate dehydrogenase (GAPDH) is shown as a protein loading control. p-S6K1(T389), p-rpS6(S240/244), and p4EBP1(T37/46) protein amounts were quantified relative to the respective total S6K1, rpS6, and 4EBP1 protein amounts by densitometry (*n* = 10 to 12 animals per group). Data are means ± standard deviations of the means. *y*-axis data represent arbitrary units. Asterisks are used to denote significance as follows: *, *P* < 0.05; **, *P* < 0.01; ***, *P* < 0.001; ****, *P* < 0.0001. Pound signs are used to denote significance as follows: #, *P* < 0.05 (by unpaired Student's *t* test).

Perturbation of mTORC1 pathway activity was assessed in tibialis anterior (TA), plantaris, and gastrocnemius muscles. Western blot analyses confirmed that the levels seen in the S6K1 arm of the mTORC1 signaling pathway were elevated in samples from multiple muscle groups of aged vehicle-treated animals relative to young adults (Fig. 2). Both LD RAD001 treatment and HD RAD001 treatment significantly reduced phosphorylation of S6K1, a direct downstream target of mTORC1, in the muscles of old rats relative to vehicle-treated animals of the same age (Fig. 2A and C). Phosphorylated S6K1 was undetectable in plantaris muscles from all treatment groups. However, phosphorylation of rpS6, a direct downstream target of S6K1, was also reduced in all muscles with both doses of RAD001 treatment compared to vehicle in old rats (Fig. 2). 4EBP1, another direct target of mTORC1, showed a partial reduction in its phosphorylation with LD RAD001, but the level was significantly reduced with the HD in TA and gastrocnemius muscles (Fig. 2A and C). Phosphorylation of 4EBP1 was significantly decreased at both doses of RAD001 in plantaris muscles (Fig. 2B). These data confirm that while HD RAD001 treatment can almost completely suppress the mTORC1 pathway, the relatively low dose of the rapalog used in the present study was also sufficient to inhibit mTORC1 signaling in aged skeletal muscle.

### Skeletal muscle mass is increased in sarcopenic rats treated with the rapalog RAD001.

**FIG 3:**
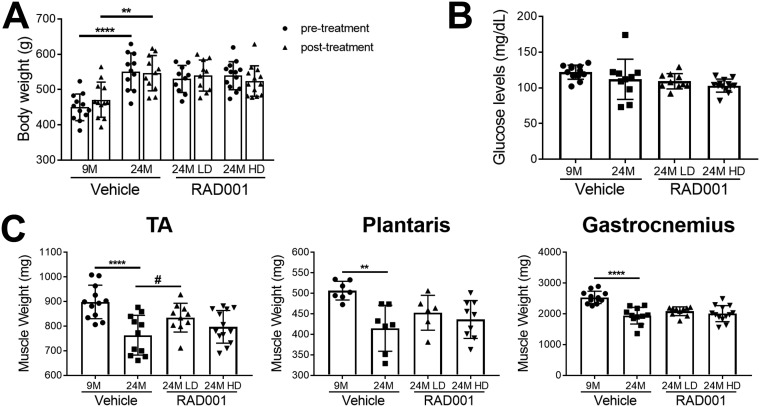
Low-dose RAD001 treatment increases mass of select skeletal muscles. (A) Total body weights of 9- and 24-month-old rats treated with vehicle and 24-month-old rats treated with 0.15 mg/kg (LD) or 0.5 mg/kg (HD) RAD001 (*n* = 11 to 13 animals per group). (B) Blood glucose levels measured at the end of study (*n* = 10 to 13 animals per group). (C) Weights of tibialis anterior (TA), plantaris, and gastrocnemius muscles from the animals described in the panel A legend following treatment (*n* = 6 to 13 animals). Data are means ± standard deviations of the means. Asterisks are used to denote significance as follows: **, *P* < 0.01; ****, *P* < 0.0001. Pound signs are used to denote significance as follows: #, *P* < 0.05 (by unpaired Student’s *t* test).

Chronic activation of the mTORC1 pathway by muscle-specific deletion of *Tsc1*, a negative regulator of mTORC1, has been shown to cause late-onset myopathy with muscle atrophy in young adult mice (47). Inhibition of mTORC1 activity using rapamycin was able to reverse the observed pathological changes and normalize muscle mass in these animals (47). To determine if we were able to ameliorate age-related muscle loss with rapalog treatment, we measured the wet weights of TA, plantaris, and gastrocnemius muscles. Consistent with previous data, all muscles from 24-month-old vehicle-treated rats had considerably reduced mass compared to 9-month-old rats (Fig. 3C). RAD001 treatment did not lead to further atrophy in any of these muscles (Fig. 3C). In contrast, rapalog treatment appeared to be protective for aged animals and reduced extensive muscle mass loss. Plantaris and TA muscles showed a surprising increase in mass, particularly with LD RAD001 treatment, with the TA muscle mass being significantly increased compared to that seen with vehicle-treated animals at this dose (Fig. 3C). Of note, overall body weight was unaffected by either dose of RAD001, as shown by comparative body measurements pre- and posttreatment (Fig. 3A). Blood glucose levels were also comparable between all aged groups (Fig. 3B). Thus, our data provide strong evidence that, when administered to sarcopenic rats, rapalog treatment is not detrimental to muscle mass. Rather, especially when given at a clinically relevant low dose, it enables animals to maintain muscle.

### Partial mTORC1 inhibition reverses molecular changes associated with sarcopenia.

We previously reported on age-related gene expression changes that helped to demonstrate the molecular pathogenesis of sarcopenia (46). Those data revealed the transcriptional upregulation of several pathways, including pathways related to innate inflammation and senescence, cellular processes counterregulated by partial mTORC1 inhibition. Because RAD001-treated animals displayed a remarkable maintenance of mass with partial mTORC1 inhibition, we sought to determine the molecular changes that could account for this.

**FIG 4:**
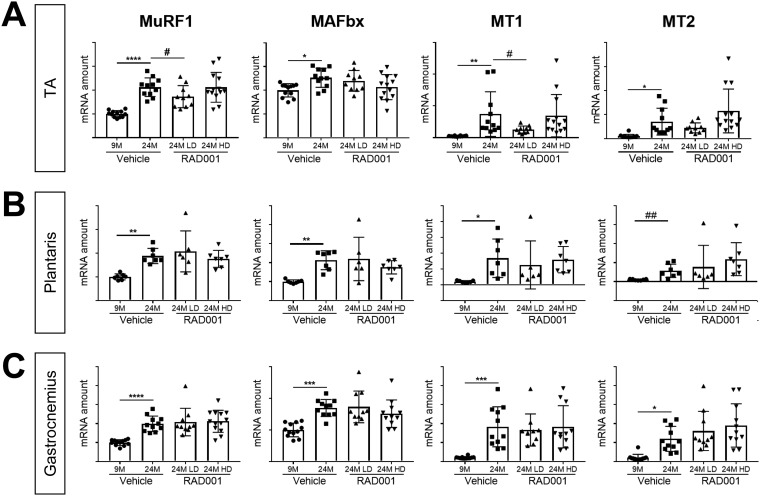
Gene expression of atrophy markers is modulated by rapalog treatment. mRNA amounts are indicated for MuRF1, MaFbx, MT1, and MT2 in tibialis anterior muscles (A), plantaris muscles (B), and gastrocnemius muscles (C) of 9- and 24-month-old rats treated with vehicle and 24-month-old rats treated with 0.15 mg/kg (LD) or 0.5 mg/kg (HD) RAD001 (*n* = 6 to 13 animals per group). mRNA amounts were standardized to geometric means of results from the TBP gene and the Vps26a gene, used as reference genes in panels. Data are means ± standard deviations of the means. Asterisks are used to denote significance as follows: *, *P* < 0.05; **, *P* < 0.01; ***, *P* < 0.001; ****, *P* < 0.0001. Pound signs are used to denote significance as follows: #, *P* < 0.05; ##, *P* < 0.01 (by unpaired Student’s *t* test). *y*-axis data represent arbitrary units.

The E3 ubiquitin ligases MuRF1 and MAFbx are important regulators of muscle atrophy that are transcriptionally upregulated under atrophic conditions (1, 33). Additionally, the level of expression of the metallothioneins MT1 and MT2 increases during atrophy and is elevated in sarcopenic muscle (48). Genetic silencing of these genes promotes muscle hypertrophy *in vivo* (48). Gene expression of these atrophy markers was analyzed in young and old TA, plantaris, and gastrocnemius muscles treated with vehicle and in old muscles treated with RAD001 (Fig. 4). All old muscles treated with vehicle had significantly higher levels of MuRF1, MAFbx, MT1, and MT2 mRNA than were seen in young muscles (Fig. 4).

Of the muscles analyzed, RAD001 treatment had the most significant impact in the TA muscle. Low-dose (LD) RAD001 reduced MuRF1 and MT1 gene expression levels in old muscles (Fig. 4A). The levels of MT2 in the TA remained unchanged with treatment (Fig. 4A). In contrast to MuRF1 and MT1 perturbation by the rapalog, MAFbx expression was not regulated (Fig. 4A). Interestingly, despite the trending increase of mass in plantaris muscle with LD RAD001, mRNA expression of the atrophy markers was not altered by treatment (Fig. 4B). Gene expression levels were also unaffected in gastrocnemius muscles from aged animals following treatment (Fig. 4C). HD RAD001 treatment was unable to downregulate expression of any of the atrophy genes, in any of the examined muscles (Fig. 4).

Together with the observed increase in muscle mass (Fig. 3C), these data confirmed that at the molecular level, mTORC1 inhibition by RAD001 does not further induce atrophy in sarcopenic muscle. Furthermore, in select muscles, when administered at a low dose, RAD001 can prevent additional muscle loss and can suppress the expression of putative atrophy genes.

**FIG 5:**
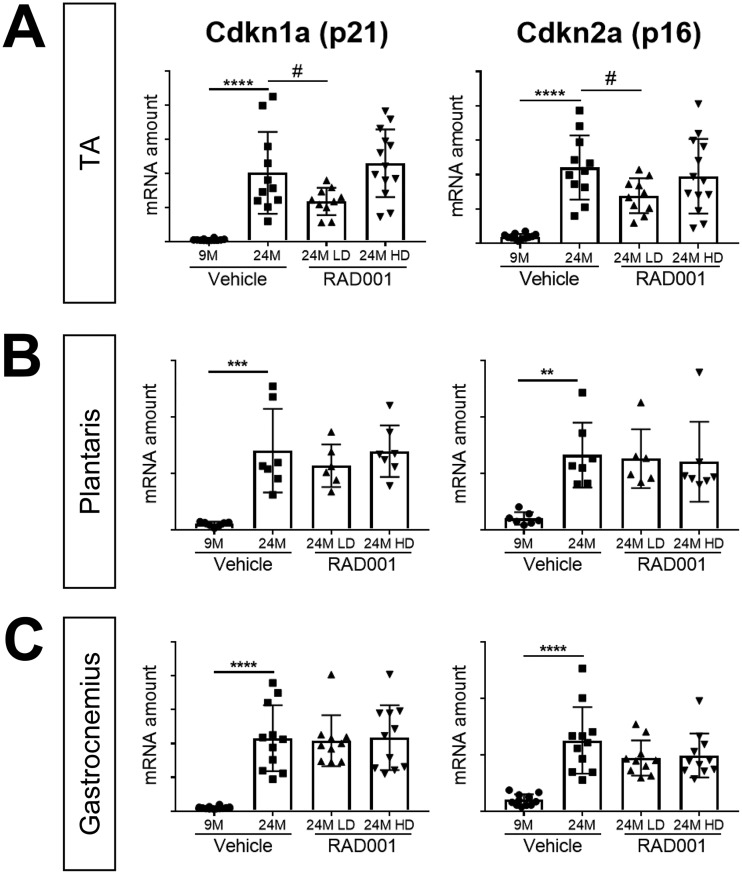
mTORC1 inhibition blunts molecular changes associated with sarcopenia. mRNA amounts are indicated for Cdkn1a and Cdkn2a in tibialis anterior (A), plantaris (B), and gastrocnemius (C) muscles of 9- and 24-month-old rats treated with vehicle and 24-month-old rats treated with 0.15 mg/kg (LD) or 0.5 mg/kg (HD) RAD001 (*n* = 6 to 13 animals per group). mRNA amounts were standardized to geometric means of results from the TBP gene and the Vps26a gene, used as reference genes. Data are means ± standard deviations of the means. Asterisks are used to denote significance as follows: **, *P* < 0.01; ***, *P* < 0.001; ****, *P* < 0.0001. Pound signs are used to denote significance as follows: #, *P* < 0.05 (by unpaired Student’s *t* test). *y*-axis data represent arbitrary units.

The onset of senescence with age is associated with the inability to efficiently repair and recover muscle, a factor contributing to the progressive decline in muscle mass in sarcopenia. Cell cycle proteins Cdkn1a (p21) and Cdkn2a (p16) are known cellular senescence markers that are upregulated with age in several tissues, including skeletal muscle (46, 49, 50). Relative to the levels seen in 9-month-old rats, Cdkn1a (p21) and Cdkn2a (p16) were both highly expressed at the mRNA level in muscles from aged vehicle-treated rats (Fig. 5). LD RAD001, but not HD, significantly reduced Cdkn1a and Cdkn2a mRNA levels in 24-month-old TA muscles compared to age-matched vehicle-treated controls (Fig. 5A). We observed no changes in the levels of expression at either dose of RAD001 in plantaris or gastrocnemius muscles (Fig. 5B and C).

### RAD001 treatment provides protection from age-associated signs of denervation.

**FIG 6:**
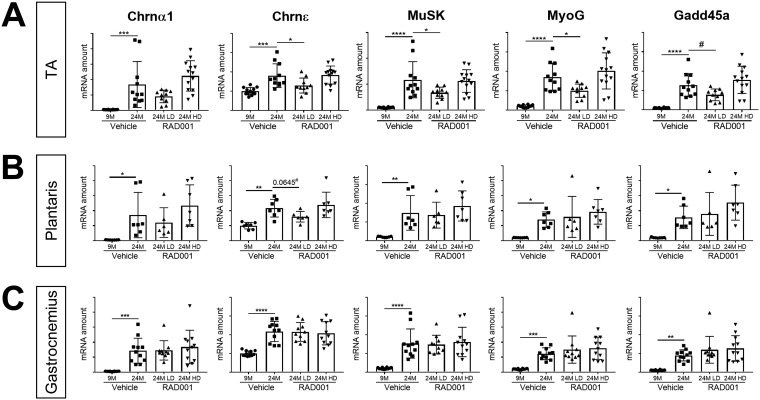
mRNA amounts of denervation markers are reduced by low-dose rapalog treatment. mRNA amounts are indicated for Chrnα1, Chrnε, Musk, MyoG, and Gadd45a in tibialis anterior (A), plantaris (B), and gastrocnemius (C) muscles of 9- and 24-month-old rats treated with vehicle and 24-month-old rats treated with 0.15 mg/kg (LD) or 0.5 mg/kg (HD) RAD001 (*n* = 6 to 13 animals per group). mRNA amounts were standardized to geometric means of results from the TBP gene and the Vps26a gene, used as reference. Data are means ± standard deviations of the means. Asterisks are used to denote significance as follows: *, *P* < 0.05; **, *P* < 0.01; ***, *P* < 0.001; ****, *P* < 0.0001. Pound signs are used to denote significance as follows: #, *P* < 0.05 (by unpaired Student’s *t* test). *y*-axis data represent arbitrary units.

Along with the deterioration of muscle tissue, the breakdown of the neuromuscular junction (NMJ) also contributes to muscle weakness; degeneration of the NMJ occurs with aging in rodents (51–53). Previous work identified the transcriptional perturbation of several genes associated with functional denervation (i.e., detachment of nerves from myofibers) in rat sarcopenic muscle (46). We therefore investigated whether mTORC1 inhibition could reverse these transcriptional changes. The expression levels of a panel of select genes, namely, the Chrnα1 and Chrnε genes, which encode subunits of the acetylcholine receptor, and the genes for MuSK, myogenin (MyoG), and Gadd45a, known to be markers of functional denervation (46), were determined by quantitative reverse transcription-PCR (RT-qPCR). In agreement with our previous observations, all of these genes were significantly upregulated in muscles from vehicle-treated 24-month-old animals compared to 9-month-old controls (Fig. 6).

For the most part, the expression levels of these genes remained unchanged in gastrocnemius and plantaris muscles following RAD001 treatment, although we observed a trend toward significance in reduction of Chrnε mRNA levels with LD RAD001 in the plantaris (Fig. 5B and C). Interestingly, in the TA muscle, treatment of aged animals with LD RAD001 reduced the transcriptional upregulation of these denervation-associated gene markers compared to vehicle-treated age-matched controls (Fig. 5A). These data suggest that suppressing the mTORC1 pathway in aged animals could be protective against age-associated denervation, at least in the TA muscle.

### Low-dose rapalog treatment may reestablish autophagy in sarcopenic muscle.

Autophagy declines with age, and studies have shown that this may result in a detrimental atrophic response contributing to the loss of muscle mass in sarcopenia (42, 43, 54, 55). Prior data suggest that autophagy induction via exercise or caloric restriction may provide protection from sarcopenia (56). Recent publications have identified the AMP-activated protein kinase (AMPKα) energy-sensing pathway as a positive modulator of autophagy, acting via direct phosphorylation of Unc-51-like autophagy activating kinase 1 (ULK1) to initiate autophagy (38, 57). Muscle-specific knockout of AMPKα in adult mice resulted in premature muscle deficiencies similar to those seen in sarcopenic mice (58). Of note, mTORC1 is a suppressor of autophagy; thus, we sought to determine whether RAD001 inhibition of mTORC1 would be sufficient to restore autophagy signaling in sarcopenic muscle.

**FIG 7:**
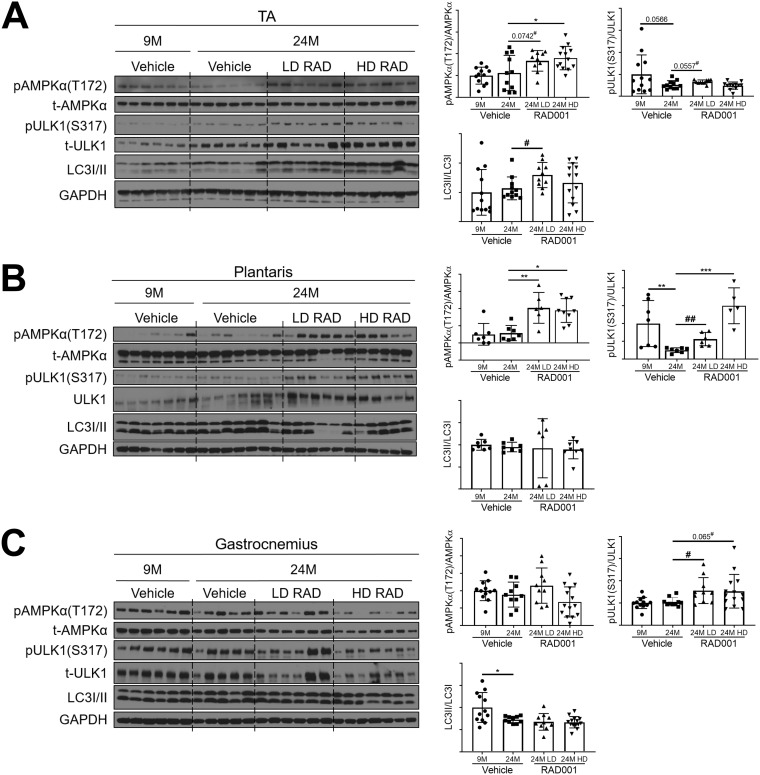
Low-dose rapalog treatment may reestablish autophagy in sarcopenic rats. Representative immunoblots are shown for phosphorylated (p) and total (t) protein for AMPKα, ULK1, and LC3I/II in tibialis anterior (A), plantaris (B), and gastrocnemius (C) muscles of 9- and 24-month-old rats treated with vehicle and 24-month-old rats treated with 0.15 mg/kg (LD) or 0.5 mg/kg (HD) RAD001. Glyceraldehyde-3-phosphate dehydrogenase (GAPDH) is shown as a protein loading control. Phosphorylated protein amounts were quantified relative to the respective total protein amounts by densitometry (*n* = 6 to 13 animals per group). LC3II protein amounts were quantified as a ratio relative to LC3I protein amounts. Data are means ± standard deviations of the means. Asterisks are used to denote significance as follows: *, *P* < 0.05; **, *P* < 0.01; ***, *P* < 0.001. Pound signs are used to denote significance as follows: #, *P* < 0.05; ##, *P* < 0.01 (by unpaired Student’s *t* test). *y*-axis data represent arbitrary units.

For all examined muscle types, phosphorylated AMPKα levels were similar between young and old vehicle-treated groups (Fig. 7). However, relative to the levels seen with old vehicle-treated animals, there was a significant increase in the phosphorylation of AMPKα in TA and plantaris muscles of rats that received low or high doses of RAD001 (Fig. 7A and B). Consistent with the increased pAMPKα levels, LD-rapalog-treated TA muscles and plantaris muscles at both doses showed greater phosphorylation of ULK1 at the AMPKα-specific phosphorylation site, serine 317, than the muscles of vehicle-treated age-matched controls (Fig. 7A and B).

Levels of the autophagosomal marker LC3II, an indicator of active autophagy, were markedly upregulated with LD RAD001 treatment in the TA muscle (Fig. 7A) and in the plantaris muscles (Fig. 7B) in four of six low-dose-treated rats. Interestingly, HD RAD001 treatment did not significantly change LC3II levels in the TA or plantaris (Fig. 7A and B). Gastrocnemius muscles from the RAD001-treated rats also displayed elevated levels of phosphorylated ULK1, although in the absence of a notable increase in phosphorylated AMPKα (Fig. 7C). However, this did not translate to an increase in LC3II levels (Fig. 7C), suggesting that autophagic function was not restored in gastrocnemius muscles.

### Improved muscle morphology in sarcopenic rats treated with a low dose of RAD001.

**FIG 8:**
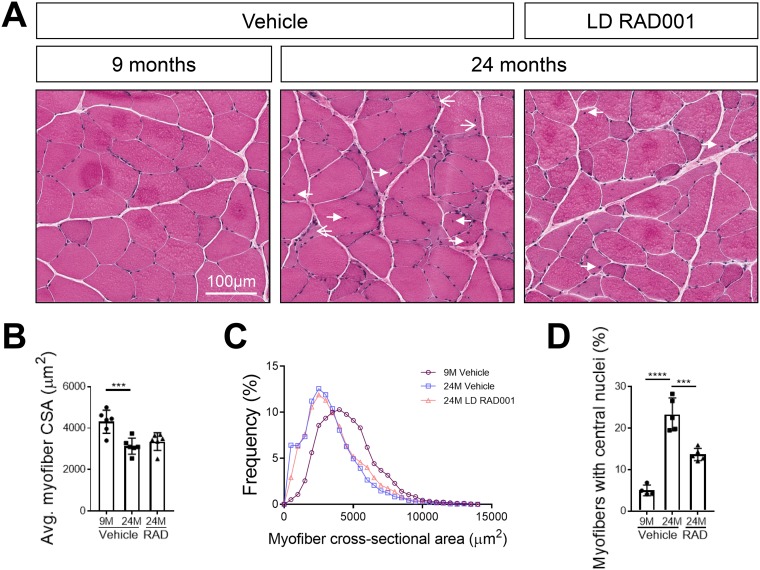
Improved skeletal muscle morphology with low-dose RAD001 treatment. (A) Representative images of transverse sections of plantaris muscles stained with H&E from 9- and 24-month-old rats treated with vehicle (*n* = 4 and *n* = 5 animals per group, respectively) and 24-month-old rats treated with 0.15 mg/kg (LD) RAD001 (*n* = 5 animals). Arrows with open arrowheads indicate misshaped, flattened myofibers. Arrows with filled arrowheads indicate central nuclei. (B) Average myofiber cross-sectional area (CSA) in plantaris muscles (*n* > 1,200 myofibers assessed per animal). (C) Histogram depicting the distribution of myofiber cross-sectional areas from data shown in panel B. Myofiber cross-sectional area frequencies are shown as a percentage of total myofibers in the given treatment group. (D) Quantification of myofibers with central nuclei in plantaris muscles. Myofibers with central nuclei are shown as a percentage of total myofibers (*n* > 1,200 myofibers assessed per animal). Data are means ± standard deviations of the means. Asterisks are used to denote significance as follows: ***, *P* < 0.001; ****, *P* < 0.0001.

With LD RAD001 treatment but not HD RAD001 treatment, TA and plantaris muscles from aged animals had increased muscle mass, in conjunction with positive molecular changes that may have contributed to these observations. Changes in muscle mass often reflect morphological alterations in tissue. We performed histological analysis on hematoxylin and eosin (H&E)-stained plantaris muscle cross sections, focusing specifically on muscle from LD RAD001-treated animals. Tissue from 9-month-old rats had normal morphology, typical of healthy muscle (Fig. 8A). In contrast, we detected several indicators of distressed muscle in aged animals that received vehicle only. Consistent with the observed trend of increased plantaris muscle mass in LD RAD001-treated rats, the average myofiber cross-sectional area tended to increase relative to the results seen with vehicle treatment (Fig. 8B). However, the most obvious change was a reduced frequency of very small, misshaped myofibers, a phenotype associated with muscle atrophy (Fig. 8A and C). Roughly 6% of myofibers from muscle from vehicle-treated animals fell within the smallest range of cross-sectional area compared to 3% in the LD RAD001 cohort (Fig. 8C). Moreover, about 23% of myofibers from vehicle-treated 24-month-old muscles presented with central nuclei, indicative of prior degeneration and ongoing regeneration (Fig. 8A and D). There was a striking reduction in the number of myofibers with central nuclei in 24-month-old plantaris muscles treated with RAD001 compared with aged-matched muscles from rats treated with vehicle (Fig. 8A and D). Taken together, these data show that low-dose rapalog treatment for 6 weeks can counteract age-related morphopathological changes in sarcopenic skeletal muscle—especially signs of degeneration requiring regeneration—as measured by the presence of central nuclei.

## DISCUSSION

Age-associated diseases comprise many of the most serious conditions afflicting human beings, including sarcopenia (and frailty), cancer, heart disease, Alzheimer's disease, and chronic kidney disease. The mTORC1 inhibitor rapamycin and its analogs (rapalogs) have been shown to extend life span (12, 14) and to delay many of these age-related conditions (9–11). These findings have even been extended to human beings, where treatment with a rapalog reversed immune senescence and increased responses to vaccines; the response to vaccines normally declines with age (16). One subject which caused some concern when it came to giving mTORC1 inhibitors to aged subjects was the skeletal muscle, since mTORC1 activation mediates protein synthesis (59) and mTORC1 inhibition blocks load-dependent hypertrophy (59). However, when we examined mTORC1 signaling in skeletal muscles in rats at ages where sarcopenia occurs (46), we were surprised to see that the level of signaling had increased rather than decreased—there was an age-related increase in the phosphorylation of S6K1 and rpS6, readouts of mTORC1 activity. Coincident with elevated mTORC1 signaling, there was a progressive decrease in skeletal muscle mass. These findings established at least that activation of mTORC1 was coincident with atrophy and therefore was not sufficient to prevent muscle loss under sarcopenic conditions. We therefore asked whether counterregulating this age-associated increase in mTORC1 signaling may perhaps be beneficial for skeletal muscle, and thus we treated aged rats for 6 weeks with a rapalog, RAD001, at a clinically relevant low dose and a relatively high dose. This low-dose strategy was reverse-translated based on a human study that showed immune cell rejuvenation after 6 weeks of RAD001 treatment (16). Treatment with a similar low dose of the rapalog RAD001, although with a distinct dosing regimen (intermittent dosing), had recently been shown to delay age-related changes in the kidney (17).

Our study demonstrated that mTORC1 activity is broadly sustained in sarcopenia but that there are differential responses to its inhibition by RAD001 in sarcopenic muscle. Following treatment, skeletal muscle mass was unchanged in the gastrocnemius muscles, but we were surprised to see that muscle mass increased rather than decreased in the TA muscles, with a moderate effect on the plantaris muscles, as a result of partial mTORC1 inhibition with a low dose of RAD001. This was not due to adverse events such as edema, and animals maintained body weight during treatment. Complete inhibition of the pathway by the use of a higher dose of RAD001 had no effect on muscle mass. Examination of individual myofibers from tissues with maintained muscle mass showed a trend toward increased cross-sectional area; the very small atrophic fibers that are found with age were in particular absent with low-dose rapalog treatment. With age, there is a dramatic increase in muscle fibers with central nuclei—a sign of muscle undergoing degeneration followed by regeneration. Treatment with the rapalog for 6 weeks decreased the number of myofibers with central nuclei by almost half, which is an indication that there was less functional degeneration and thus less of a need for subsequent regeneration. In line with this, there were also signs that functional denervation occurred with age; this impression was bolstered at the molecular level by the demonstration that levels of gene markers associated with denervation, including the MuSK gene and several of the acetylcholine receptor genes, increased with age in all muscle groups—consistent with a prior report (46). Some of these denervation markers were counterregulated by the rapalog in the TA and plantaris muscles but not in the gastrocnemius muscles, suggesting that rapalog treatment may prevent functional denervation, providing an additional mechanism for preservation of muscle mass. On the basis of these data, the effects of RAD001 treatment on muscle function warrant further investigation.

Rapalog treatment decreased mTORC1 signaling in all muscle groups as detected by dephosphorylation of S6K1 and its downstream target rpS6, as well as of 4EBP1. The levels of counterregulation of sarcopenia-associated molecular markers in response to inhibition differed between muscles. However, in muscles showing significant increases in mass, mRNA levels of the putative atrophy marker MuRF1 were considerably reduced coincident with mTORC1 inhibition. In addition to MuRF1, the metallothionein MT1 was downregulated. We had previously shown that this is a high-fidelity marker of atrophy and that knocking out the MT genes in mice causes muscle hypertrophy (48). This finding as well is consistent with the increase in mass observed in the present study and provides a further mechanistic rationale. As for the levels of senescence markers p16 and p21, they were elevated in aged muscle compared to young muscle and then reapproached the level seen with younger muscle following rapalog treatment, consistent with what had been shown previously in geriatric satellite cells (49, 60). This reversal of senescent markers suggested the possibility of improvement of the satellite cell function necessary for muscle homeostasis, reflected by the positive morphological changes that we observed in low-dose-rapalog-treated muscles. Strangely, although the plantaris muscles displayed a moderate improvement in mass and reversal of pathological changes, expression levels of atrophy and senescent markers remained unchanged in this muscle. Given the differences in the effects observed between muscles, our data suggest that mTORC1 inhibition alone is not sufficient to maintain muscle mass in sarcopenia.

Autophagy induction may also be required to promote muscle health. mTORC1 is a negative regulator of autophagy through its suppression of ULK1. Furthermore, muscle-specific deletion of AMPKα impairs autophagy in aged tissue, causing a premature pathological phenotype similar to that in sarcopenic muscle (58). Interestingly, in our study, only the muscle groups that showed an increase in mass with rapalog treatment had restored autophagy, possibly via activation of the AMPKα pathway, as indicated by the presence of the autophagosomal marker LC3II. This effect was observed with partial mTORC1 inhibition only after low-dose RAD001 treatment, in comparison to the stronger inhibition induced by high doses of RAD001. It appears then that the amelioration of the sarcopenic phenotype requires a careful equilibrium between the inhibition of overactivated anabolic pathways such as the mTORC1 pathway and boosting the activity of catabolic pathways such as those governed by AMPKα. Reestablishing a basal level of autophagy may serve to recycle macromolecules for the support of cell growth and clearance of dysfunctional organelles. Other catabolic functions downstream of AMPKα may also contribute to the maintenance of muscle mass and present an interesting avenue for further study.

In summary, mTORC1 signaling is hyperactivated in aged muscle, and this apparently contributes to sarcopenia. Partial rather than complete inhibition of mTORC1 signaling has beneficial effects in sarcopenic muscle since inhibition of this signaling can increase muscle mass, albeit not in all muscles. Reestablishment of autophagy via enhanced AMPKα signaling is additionally required for the maintenance of muscle health. The inhibition of denervation and senescence markers and the subsequent decline in atrophy markers give a further therapeutic rationale for treating aged sarcopenic patients with an mTORC1 inhibitor.

## MATERIALS AND METHODS

### Animal maintenance and RAD001 treatment.

Male Sprague-Dawley rats were obtained from Envigo (Indianapolis, IN) and were housed at their facility under specific-pathogen-free (SPF) conditions until the rats reached appropriate age. When transferred to the Novartis Institutes for Biomedical Research (NIBR) (Cambridge, MA) facility, rats continued to be maintained under SPF conditions, with regulated temperature and light cycle (22°C, 12-h light/12-h dark cycle [lights on at 0600 h/lights off at 1800 h]) and unrestricted access to food (2014 Teklad global 14% protein diet; Envigo) and water. Animals were acclimated for a minimum of 4 weeks before being used for experiments. For aging time course studies, rats ranging from 6 to 27 months of age (*n* = 6 to 8 animals/group) were fasted from 0600 h to 1200 h (during the light-on cycle) before being anesthetized and euthanized for end-of-study analysis. Gastrocnemius muscles were collected for molecular analysis. For other studies, RAD001 (Novartis) was prepared as a microemulsion preconcentrate at 2% (wt/vol). Prior to dosing, it was diluted to a working concentration in water. The vehicle control consisted of microemulsion preconcentrate (equivalent to one dose) diluted in water. At 22 months, animals were dosed *per os* daily for 6 weeks with either RAD001 or vehicle. In parallel, rats aged 7 months received vehicle as young-adult controls. Four hours after the last dose of RAD001 or vehicle, rats were anesthetized with 3.5% isofluorane and were euthanized by exsanguination and thoracotomy. Gastrocnemius, plantaris, and tibialis anterior (TA) muscles were collected and weighed; TA, gastrocnemius, and plantaris muscles were processed as described below for further assessment. All animal studies were done in accordance with institutional guidelines for the care and use of laboratory animals as approved by the Institutional Animal Care and Use Committee (IACUC) of the Novartis Institutes of Biomedical Research, Cambridge, MA.

### Protein extraction and Western blot analysis.

For protein extraction, snap-frozen muscles were pulverized in liquid nitrogen by the use of a mortar and pestle to a fine powder. Approximately 30 mg of tissue powder was homogenized in Meso Scale Discovery (MSD) lysis buffer (catalog no. R60TX; Meso Scale Discovery) supplemented with protease and phosphatase inhibitor cocktail (Thermo Fisher Scientific, MA). Following a 30-min incubation at 4°C with agitation, protein lysates containing the cytoplasmic fraction were collected via microcentrifugation. Protein concentrations were determined by bicinchoninic acid (BCA) protein assay (Thermo Fisher Scientific, MA), prior to Western blot analysis. Diluted proteins were separated by sodium dodecyl sulfate polyacrylamide gel electrophoresis (SDS-PAGE) on a Criterion TGX Precast Midi Protein gel (Bio-Rad, CA) (4% to 20% gradient) and subsequently transferred onto nitrocellulose membranes (Bio-Rad, CA) by the use of a Trans Turbo Blot system (Bio-Rad). Membranes were blocked in 5% milk–Tris-buffered saline with Tween 20 (TBST) for 1 h at room temperature and were then incubated with primary antibodies overnight at 4°C. After three washes in TBST, membranes were incubated in the appropriate horseradish peroxidase (HRP)-conjugated secondary antibodies (Cell Signaling Technologies) for 1 h at room temperature. The following primary antibodies were used: anti-GAPDH (anti-glyceraldehyde-3-phosphate dehydrogenase) (catalog no. 5174), anti-rpS6 (catalog no. 2217), anti-p-rpS6(S240/244) (catalog no. 2215), anti-S6K1 (catalog no. 2708), anti-pS6K1(T389) (catalog no. 9234), anti-4EBP1 (catalog no. 9644), anti-p-4EBP1(T37/46) (catalog no. 2855), anti-AMPKα1 (catalog no. 2532), anti-p-AMPKα1(T172) (catalog no. 2535), anti-ULK1 (catalog no. 8054), anti-p-ULK1(S317) (catalog no. 12753), and LC3I/II (catalog no. 4108), all from Cell Signaling Technologies. Anti-rabbit and anti-mouse IgG HRP-conjugated secondary antibodies were also from Cell Signaling Technologies. Densitometric analysis was performed using Fiji 1.51n software.

### Muscle cryosectioning.

Plantaris muscles were embedded in OCT (Tissue-Tek) and flash frozen in liquid nitrogen chilled in 2-methylbutane (Fisher Scientific). Muscles were sectioned transversely with a Leica CM3050 S microtome and 10-μm-thick sections were collected for hematoxylin and eosin (H&E) staining and immunohistochemistry.

### Hematoxylin and eosin staining.

Muscle sections were fixed in 4% paraformaldehyde on ice for 10 min and rinsed briefly with water five times. H&E staining was done using a Tissue-Tek Prisma automated slide stainer (Sakura Finetek). Images were captured using a Aperio ScanscopeAT scanner (Leica Biosystems) and were used to determine morphological changes, including the incidence of central nuclei.

### Immunohistochemistry.

Myofiber cross-sectional areas were measured on muscle cross sections immunostained with the antilaminin antibody. Briefly, tissue sections were fixed in 4% paraformaldehyde on ice for 10 min and were then washed in 1× phosphate-buffered saline (PBS) prior to permeabilization in 0.3% Triton X-100–PBS. Nonspecific sites were blocked in 16% goat serum diluted in 0.01% Triton X-100–PBS (blocking buffer) for 1 h at room temperature. Sections were incubated in antilaminin (Sigma-Aldrich, L9393) antibody diluted at 1:1,000 in blocking buffer overnight at 4°C. Primary antibody was detected after 1 h of incubation with Alexa Fluor-conjugated goat anti-rabbit secondary antibody (Life Technologies; catalog no. A-11072) diluted in blocking buffer. Following a series of washes in 0.01% Triton X-100–PBS, slides were mounted with Fluoromount-G (SouthernBiotech). Images were captured using a VS120 virtual slide microscope (Olympus) at ×20 magnification.

### RNA extraction, cDNA synthesis, and quantitative RT-PCR (RT-qPCR).

Approximately 30 mg of ground tissue powder was processed using a miRNeasy microkit (Qiagen) according to the manufacturer’s protocol. RNA concentrations were quantified by the use of a NanoDrop spectrophotometer (NanoDrop Technologies), and quality was confirmed by the optical density at 260 nm (OD_260_)/OD_280_ absorption ratio (>1.8). Following the manufacturer’s protocol, cDNA was synthesized from 1 μg of RNA using a High Capacity cDNA reverse transcription kit (Applied Biosystems by Thermo Fisher Scientific). cDNA was diluted 1:10 in Ultra Pure distilled RNase-free water (Invitrogen) prior to being used for further analysis steps. Standard TaqMan gene expression master mix (Applied Biosystems by Thermo Fisher Scientific) was used for all RT-qPCRs, and samples were run using a 384-well optical plate format. Reactions were performed using a ViiA 7 RT-qPCR system (Life Technologies), and data were analyzed by the threshold cycle (ΔΔ*C_T_*) method. TaqMan probes were optimized by Applied Biosystems and consisted of the following: TATA box binding protein (TBP) (Rn01455646_m1), Vps26a (Rn01433541_m1), MurF1 (Rn01639111_m1), MaFbx (Rn00591730_m1), Mt1 (Rn00821759_g1), Mt2A (Rn01536588_g1), Cdkn1a (Rn00589996_m1), Cdkn2a (Rn00580664_m1), Chrna1 (Rn01278033_m1), Chrne (Rn00567899_m1), MuSK (Rn00579211_m1), myogenin (Rn00567418_m1), and Gadd45a (Rn01425130_g1).

### Statistical analysis.

Statistical significance was determined using GraphPad Prism 7.04 software by a one-way analysis of variance (ANOVA) followed by Dunnett’s multiple-comparison tests. Means of data from all groups were compared to the mean of the data from the aged, vehicle-treated group, except where otherwise specified. All data are presented as means with standard deviations.
